# Guidance on the interpretation of faecal calprotectin levels in children

**DOI:** 10.1371/journal.pone.0246091

**Published:** 2021-02-11

**Authors:** Martina Orfei, Marco Gasparetto, Kai O. Hensel, Florian Zellweger, Robert B. Heuschkel, Matthias Zilbauer

**Affiliations:** 1 Department of Paediatric Gastroenterology, Hepatology and Nutrition, Cambridge University Hospitals, Cambridge, United Kingdom; 2 Department of Paediatrics, Faculty of Health, University Witten/Herdecke, Centre for Clinical and Translational Research, Witten, Germany; 3 Department of Plant Sciences, University of Cambridge, Cambridge, United Kingdom; 4 Swiss Federal Research Institute WSL, Birmensdorf, Switzerland; 5 Department of Paediatrics, University of Cambridge, Cambridge, United Kingdom; McMaster University, CANADA

## Abstract

**Background:**

Faecal calprotectin (FCP) is a powerful tool to predict inflammatory bowel disease (IBD) in patients with gastrointestinal symptoms. In the paediatric patient population, the reference value of < 50 μg/g and the influence of age on FCP levels result in a high number of redundant investigations and specialist referrals. We assessed paediatric FCP levels, their diagnostic value and corresponding referral pathways from primary and secondary care.

**Methods:**

We analysed two cohorts from a precisely defined catchment area: one consisted of all FCPs measured in this area (n = 2788). The second cohort—a subset of the first cohort—consisted of FCP values and corresponding clinical data from children who were referred for possible IBD to our department (n = 373).

**Results:**

In the first cohort, 47% of FCP levels were > 50 μg/g, 15% were ≥ 250 μg/g. Children < 1y had significantly (p < 0.001) higher FCP than older children. In the second cohort, 6.7% of children with an FCP of < 250 μg/g (or 8.6% with an FCP of < 600 μg/g) had IBD–all featured symptoms suggestive of IBD (e.g. bloody diarrhoea, nocturnal abdominal pain, weight loss) or abnormal blood tests. 76% of patients in whom raised FCP (> 50 μg/g) was the sole reason for being referred for suspected IBD did not have IBD.

**Conclusion:**

Children with an FCP < 600 μg/g and without matching symptoms suggestive of IBD are unlikely to have IBD. A higher FCP reference value may provide cost-effective improvement that could avoid redundant investigations and specialist referrals. A guideline for specialist referrals is proposed.

## Introduction

Detection of the faecal calprotectin (FCP) has been established as a highly reliable, yet non-specific marker for gastrointestinal (GI) inflammation [1–3]. The clinical application of FCP currently covers mainly two areas; (i) as a screening tool in the diagnostic work up for inflammatory bowel disease (IBD) in patients with GI symptoms [2, 4, 5] and (ii) to monitor disease activity in patients with IBD [6–9]. A reliable and valid reference value is critical for a screening test, but many laboratories still do not provide age-appropriate reference ranges. This is despite the well-documented higher FCP levels in healthy young children, especially in children aged < 6 years [10–15]. This inevitably leads to unnecessary healthcare costs, let alone potentially invasive investigations for children [16, 17].

Furthermore, an increasing number of children suffer from unspecific GI symptoms such as abdominal pain or diarrhoea. The most accurate way to assess these children for possible IBD is the right interpretation of the combination of matching symptoms suggestive of IBD, blood test results and FCP values above 250 μg/g [18, 19]. However, as we have seen now in our cohort, many doctors in primary care (General Practitioners, GPs) and secondary care (General Paediatricians) in the United Kingdom refer children to the paediatric gastroenterologist for possible IBD with a slightly elevated FCP just above 50 μg/g, regardless of matching symptoms suggestive of IBD and/or abnormal blood tests.

Studies assessing the wide use of FCP in primary and secondary care are scarce. This is despite the fact that GPs and General Paediatricians are the first port of call for children and refer a child to the paediatric gastroenterologist. As pressure on costs in health care rises, accuracy and standardisation of diagnostic testing and the correct interpretation of a test result are highly needed. The aims of this study are (1) to assess age-appropriate FCP values in children, (2) to assess thresholds of FCP used by primary and secondary care to refer children for suspected IBD to a tertiary level gastroenterology department and (3) to develop a practical guideline for the differentiated interpretation of FCP and for referrals from primary and secondary care.

## Methods

### Patient cohort and study design

We used the Cambridge University Hospitals (CUH) electronic patient record system “Epic” to compile FCP values and clinical data. “Epic” contains all FCP values that were ordered from all hospitals, general paediatricians and general practitioners of a specific, clearly defined catchment area around Cambridge. The catchment areas in the UK health system steadily cover strictly specific towns and their related hospitals, general paediatricians and GPs. Therefore, all FCP values ordered in this catchment area were analysed at the CUH biochemistry laboratory. Moreover, all children of this catchment area would be referred to our tertiary paediatric gastroenterology unit.

We analysed two cohorts of FCP values: one cohort consisted of all FCP levels analysed between October 2014 and July 2018 from children aged 0–16 years, resulting in a total of 2788 FCP values performed on 2788 children (1676 female, 1112 male, Table 1).

**Table 1:** Summary table of faecal calprotectin (FCP) measurements at Cambridge University Hospitals between October 2014 to July 2018.

Description	Number
Total FCP values measured	45‘717
Total FCP values measured in children 0-16y (incl. multiples) measurements)	3‘461
*thereof repeat measurements on same patient*	*673*
Single FCP measurements of children 0–16 y	2‘788
*thereof female children*	*1‘676*
*thereof male children*	*1‘112*
FCP values with corresponding entry on „EPIC”(= referred children)	377
Children with new diagnosis of IBD	96
Children referred by General Practitioners	216
Children referred by General Paediatricians	157
Children referred by Paediatric Gastroenterologists	4

This cohort comprises FCP results that were ordered and interpreted by GPs and Paediatricians in the defined catchment area—the majority of these patients were not referred nor assessed at our paediatric gastroenterology department. The second cohort—being a subset of the first cohort—consisted of FCP values from children who were referred from the same catchment area to our paediatric gastroenterology department by GPs or Paediatricians. Referrals were sent for possible IBD due to an elevated FCP level above 50 μg/g—with or without matching symptoms suggestive of IBD. For this analysis, we included FCP results of all referred children with complete data entries on “Epic” (including recorded referral letter and clinical characteristics like age, sex, gastrointestinal symptoms, final diagnosis, blood test results and medication such as non-steroidal anti-inflammatory drugs NSAIDs). We excluded FCP values without corresponding or complete data entry on “Epic”, FCP requested by our paediatric gastroenterology department and FCP of children with IBD diagnosed prior to October 2014.

Each referral letter was screened and the following information was obtained: by whom (GP/paediatrician/paediatric gastroenterologist) the child had been referred, blood test results previous to the referral and whether the referral was done solely because of the raised FCP (> 50 μg/g, regardless of matching symptoms suggestive of IBD) or because of GI symptoms (along with a raised FCP). We identified 373 FCP firstly measured values from 373 children who met the inclusion criteria. Thereof, 96 patients were subsequently diagnosed with IBD. All children with confirmed IBD received a diagnostic endoscopy (oesophagogastroduodenoscopy and ileocolonoscopy with biopsies) at our department to confirm a diagnosis of IBD based on the Porto criteria for diagnosing IBD in children [18].

All children with suspected IBD in our above mentioned catchment area would be referred to our paediatric gastroenterology department hence a diagnosis of IBD would only be confirmed at our institution (CUH) upon endoscopic, histological and imaging findings.

In some of the analyses we used age groups (< 1y, 1-5y, 6-14y, 15-16y) to enable the comparison of our results with previous findings [10, 12].

Despite the fact that many children had multiple FCP results, we used only the first measurement per child for statistical reasons.

### FCP assay

FCP levels were measured at CUH biochemistry laboratory using a standardised ELISA (Bühlmann fCal, Schönenbuch, Switzerland). Results were provided in μg/g with < 50 μg/g as normal reference value which was defined by the test kit provider Bühlmann at the time of the study. The maximum value for this test kit in our laboratory was reported as “> 600 μg/g”.

### Data analyses

Statistical analyses were performed in R version 3.5.0 [20]. A two-sided T-test was applied for quantitative variables and a Chi-Square-test and Welch two sample adaptation for categorical variables. Data is presented as absolute numbers, percentages, box and whisker plots with median and mean. We used median rather than mean values to calculate significance because means are more influenced by outliers and our laboratory reports very high values only as “> 600 μg/g”.

For the analysis of the receiver operating curve (ROC) and the area under the curve (AUC) we used the pROC package. To produce the ROC we fitted a logistic regression model and computed the AUC. The optimal cut-off value for FCP for diagnosing IBD was suggested by the “coords” function in the pROC package.

We analysed three different FCP cut-off levels: 50 μg/g as it is the current normal reference level; 250 μg/g as FCP levels of > 250 μg/g have been suggested as cut-off values to predict mucosal inflammation in IBD according to the European Society of Paediatric Gastroenterology, Hepatology and Nutrition (ESPGHAN) [9], and 600 μg/g as our laboratory reports very high values only as “> 600 μg/g”.

### Ethics statement

All data and samples of faecal Calprotectin used in this study were fully anonymized before we accessed them. Data contained hospital and patient identification numbers, date of birth, gender, date of sampling and value of faecal Calprotectin, name of the corresponding General Practitioner and whether a prescription for non-steroidal anti-inflammatory drugs (NSAIDs) was present or not. Data from the time frame October 2014—July 2018 were accessed through the Cambridge University Hospital’s electronic patient record system “Epic”. This study was registered as an audit with the Cambridge University Hospitals Audit Department (project number 26763956), and it was approved by the Research Ethics Committee (REC) of the Cambridge University Hospitals NHS Foundation Trust.

## Results

### Almost half of all paediatric FCP levels exceed current normal reference levels

**Fig 1:**
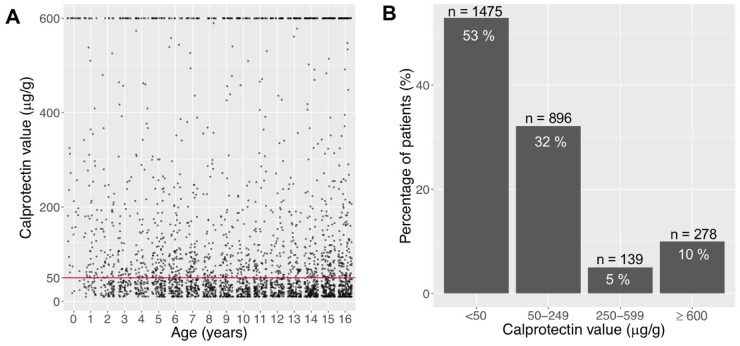
Distribution of faecal calprotectin (FCP) in the total cohort, including samples of healthy children and children with a gastrointestinal condition (e.g. IBD). **A** Scatterplot of absolute FCP distribution of 2788 samples from 2788 children aged 0-16y. **B** Percentage of all patients (n = 2788) according to FCP range. 85% of all FCP values are < 250 μg/g, 90% are < 600 μg/g.

In our first cohort (n = 2788), where all FCP levels of children from our catchment area were included, we investigated FCP levels regardless of the underlying diagnosis to overview the FCP distribution of our cohort (Fig 1A). As shown in Fig 1B, 47% exceed the current normal reference level of 50 μg/g. 10% (n = 278) of FCP lied ≥ 600 μg/g, resulting in 85% of samples below 250 μg/g.

### FCP levels are higher in children under the age of 1 year

To investigate whether young children (age < 6y) have higher FCP values than older children, we compared the FCP levels of different age groups in the first cohort (n = 2788). The average FCP level was found to be significantly (p < 0.001) higher in infants (aged < 1y) compared to all other age groups (Table 2).

**Table 2:** Summary of age-group-related comparison in FCP levels of the first cohort (n = 2788).

Age group	Number of patients	Median FCP (μg/g)	Interquartile range	p value
< 1y	n = 34	205	498	p < 0.001 compared to all other age groups
1-5y	n = 582	55	120	p < 0.001 compared to 6-14y
6-14y	n = 1598	41	80	p = 0.080 compared to 15-16y
15-16y	n = 579	47	113	p = 0.099 compared to 1-5y

FCP: Faecal Calprotectin.

Similarly, the median FCP in the age group 1-5y was significantly higher (p < 0.001) when compared to the 6-14y old group. However, the clinical FCP value was similar (41 vs. 47 μg/g). The difference in the median FCP between the age group 6-14y and 15-16y as well as between 1-5y and 15-16y was not statistically significant (p = 0.080 and 0.099, respectively, Table 2).

### Increasing FCP reference range improves diagnostic accuracy for IBD

**Fig 2:**
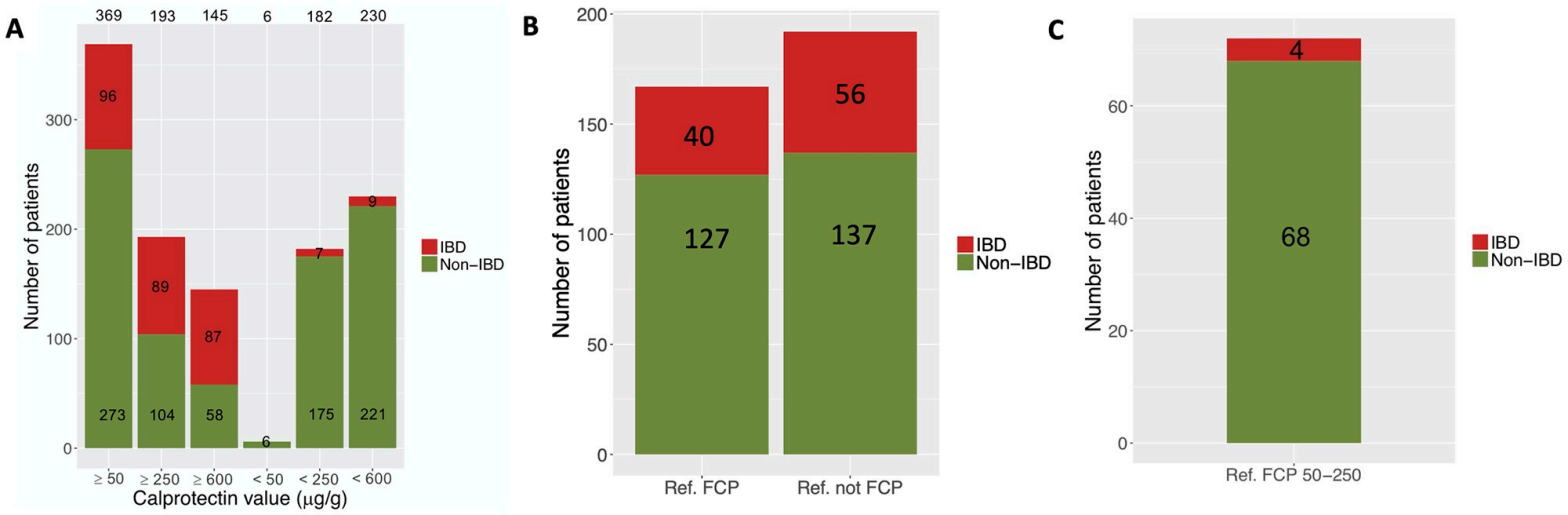
**A** Proportion of patients with newly diagnosed IBD (red) and without IBD (green) according to their FCP of </≥ 50 μg/g, </≥ 250 μg/g and </≥ 600 μg/g. The difference between IBD diagnoses in patients with FCP < and ≥ 250 μg/g as well as in those with FCP < and ≥ 600 μg/g is statistically significant (p < 0.05). **B** Proportion of patients with/without IBD who were referred due to and not due to their elevated FCP (Ref. FCP and Ref. not FCP, respectively). **C** Proportion of patients with/without IBD and referred due to their FCP of 50–250 μg/g.

Based on findings so far, we hypothesised that applying a threshold for FCP higher than 50 μg/g in children may increase the accuracy for diagnosing IBD. For these analyses, we used the second cohort (n = 373), which consists of all children referred by doctors from our precisely defined catchment area for possible IBD. None of these children were taking NSAIDs nor had a gastrointestinal polyp nor a gastrointestinal infection that could have increased the FCP. As we know the exact clinical data and the final diagnosis of the whole cohort, we could then identify all children with a confirmed diagnosis of IBD. Of the 373 patients who met the inclusion criteria, 96 had been diagnosed with IBD by upper and lower endoscopy and biopsies according to the Porto criteria for diagnosing IBD in children [18]. As shown in Fig 2A, 96% of children with an FCP level of < 250 μg/g were not diagnosed with IBD. In contrast, almost 46% of patients with FCP levels > 250 μg/g were ultimately diagnosed with IBD, with the percentage increasing to 60% in children with FCP levels > 600 μg/g (Fig 2A).

Table 3 shows that seven children with IBD (6.7% of all children with IBD) had an FCP of < 250 μg/g (i.e. 92, 101, 190, 211, 232 and 245 μg/g) and two children with IBD (1.9%) had an FCP of 250–599 μg/g (i.e. 313 and 529 μg/g).

**Table 3:** Summary of symptoms and blood test results of IBD patients with FCP < 600 μg/g (n = 9) and of IBD patients aged < 6y (n = 4).

Description of IBD patient	Symptoms	Blood test results
Patient 1, FCP < 250 μg/g	PR bleeding, nocturnal abdominal pain, watery diarrhoea	Anaemia
Patient 2, FCP < 250 μg/g	PR bleeding, watery diarrhoea	Anaemia
Patient 3, FCP < 250 μg/g	Diarrhoea, vomiting, nocturnal abdominal pain	Anaemia, raised CRP/ESR, low Albumin
Patient 4, FCP < 250 μg/g	PR bleeding, abdominal pain	Raised ESR
Patient 5, FCP < 250 μg/g	PR bleeding, abdominal pain	Raised ESR and platelets
Patient 6, FCP < 250 μg/g	PR bleeding, weight loss	Normal bloods
Patient 7, FCP < 250 μg/g	PR bleeding	Normal bloods
Patient 8, FCP 250–599 μg/g	Watery diarrhoea	Anaemia, low Albumin
Patient 9, FCP 250–599 μg/g	Weight loss, PR bleeding, abdominal pain	Raised CRP, ESR and liver enzymes
Patient 10, age < 6y	Weight loss, abdominal pain, vomiting	Anaemia, raised CRP
Patient 11, age < 6y	PR bleeding	Anaemia
Patient 12, age < 6y	PR bleeding	Raised CRP/ESR
Patient 13, age < 6y	Watery diarrhoea, weight loss	Anaemia, low Albumin

IBD inflammatory bowel disease; FCP faecal calprotectin; PR per rectum; CRP C-reactive protein; ESR erythrocyte sedimentation rate.

Importantly, all of these patients with an FCP < 600 μg/g and IBD presented initially with matching symptoms suggestive of IBD (i.e. bloody diarrhoea, weight loss, nocturnal abdominal pain) and/or at least one abnormal blood tests (i.e. elevated C-reactive protein and/or erythrocyte sedimentation rate, anaemia, raised platelets, low albumin, raised liver/bile enzymes) (Table 3). Four children with IBD (= 4% of all children with IBD) were at the age of < 6y at the diagnosis. They all showed matching symptoms suggestive of IBD (Table 3).

The ROC analysis revealed an AUC of 86.11%. The optimal FCP cut-off value was 600 μg/g. The true positive percentage (tpp)/sensitivity at this threshold was 91.58% and the false positive percentage (fpp)/(1-specificity) was 21.80%. The tpp and fpp at FCP 50 μg/g cut-off are 100% and 93.98%, and those at FCP 250 μg/g cut-off were 93.68% and 39.10%, respectively. A split of the data into different age groups revealed similar AUCs (no significant differences) and therefore supports the same thresholds.

### Specialist referrals are frequently based on FCP levels

Many referrals received by GPs and Paediatricians were exclusively based on (slightly) elevated FCP just above 50 μg/g and irrespective of the presence of matching symptoms suggestive of IBD. This means, that the child may have had abdominal pain, diarrhoea or nausea that led to an FCP measurement, in the absence of red flag signs like nocturnal abdominal pain, bloody diarrhoea or vomiting. We therefore assessed the referral pathways, the subsequent diagnosis of IBD and the reliability of FCP in the diagnosis of IBD.

In the observed study period, 373 of the 2788 children who had an FCP performed were referred for possible IBD to our paediatric gastroenterology department; 216 (58%) by GPs, 157 (42%) by General Paediatricians (Table 1). Of 373 referrals from GPs or Paediatricians, 167 (45%) were made solely because of an elevated FCP > 50 μg/g irrespective of matching symptoms suggestive of IBD (so called “red flags symptoms”, i.e. nocturnal abdominal pain, bloody diarrhoea, vomiting, weight loss) whilst 206 (55%) were made for matching symptoms suggestive of IBD and/or abnormal blood tests in addition to an elevated FCP.

Fig 2B shows the quantity of patients who were referred for possible IBD only due to their raised FCP versus those who were referred solely because of their symptoms alongside a raised FCP. We then investigated how many of them had been diagnosed with IBD afterwards. All patients included in this analysis had an FCP done prior to the referral.

Overall, 24% of all children referred due to their elevated FCP were diagnosed with IBD. 29% of the children whose primary reason for referral was not their elevated FCP were also subsequently diagnosed with IBD.

Children with an FCP of 50–250 μg/g and who were referred solely because of their raised FCP, only four patients (5.5%) were diagnosed with IBD (Fig 2C). Their FCP level was 92, 101, 232 and 245 μg/g.

### Implications for paediatric practice

**Fig 3:**
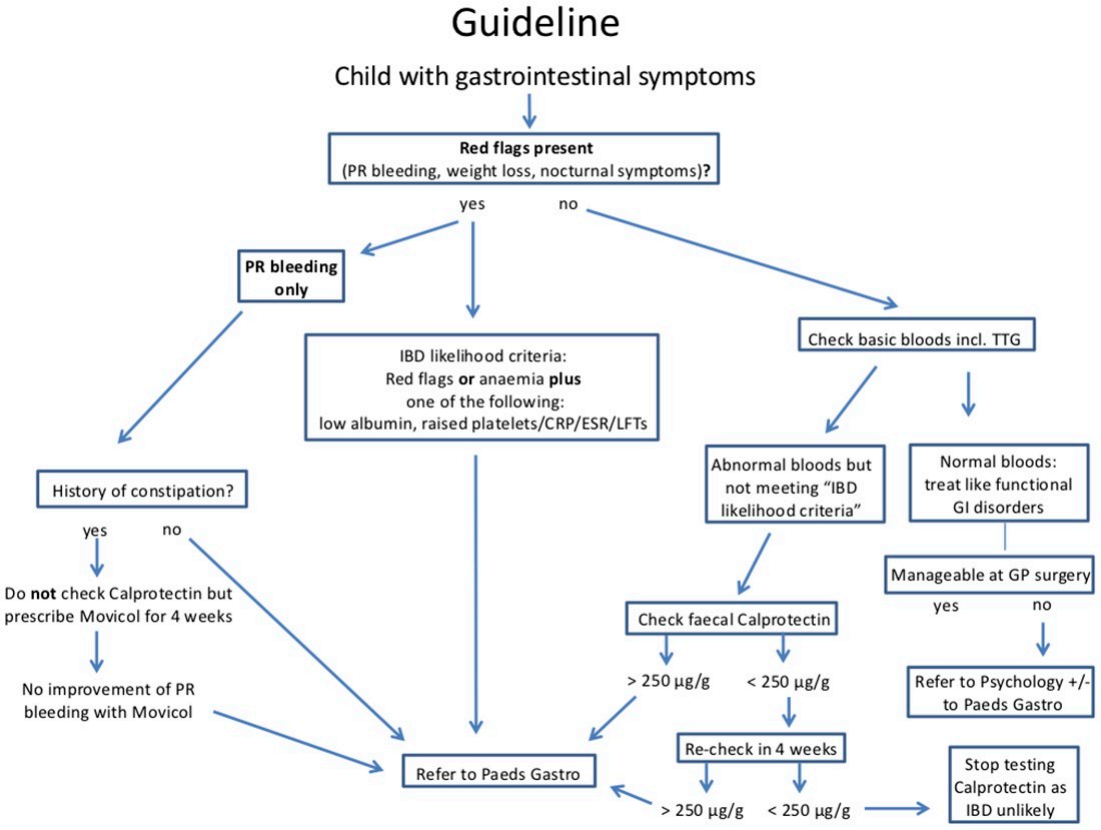
Flowchart providing a guideline on how to proceed with a child presenting with gastrointestinal symptoms in primary and secondary care. IBD Inflammatory bowel disease; PR per rectum; TTG Tissue Transglutaminase; GI gastrointestinal; CRP C-reactive protein; ESR erythrocyte sedimentation rate; LFT’s liver function tests; GP General Practitioner.

Our findings show that only a small percentage of patients (15%) have an FCP of > 250 μg/g and thereof, IBD is unlikely in a patient without matching clinical symptoms or abnormal blood test, as already shown by Van de Vijver et al. [17]. In order to support doctors in primary and secondary care in the decision making of whether FCP should be tested and a patient needs to be seen by a specialist, we suggest a flowchart as a clinical guidance (Fig 3). This flowchart is based on the findings of this study, the recommended increase of FCP cut-off to 250 μg/g by the ESPGHAN guidelines [9] and our clinical experience.

This guideline aims to reduce unnecessary investigations like endoscopies and repeated FCP measurements and to avoid redundant specialist referrals. If a child presents with matching symptoms suggestive of IBD (so-called “red flag” symptoms) and has additionally one or more abnormal blood tests (anaemia, raised platelets/CRP/ESR/liver enzymes, low albumin), the priority should be to have the child referred promptly to a paediatric gastroenterologist, who will use the FCP result to make a decision about specialist investigations. If a child only has abnormal blood test results in the absence of “red flag” symptoms, FCP should be tested and in case of a level < 250 μg/g in two separate samples, IBD will be unlikely and no further investigations should be performed. Based on clinical practice we recommend to test FCP twice in primary and secondary care, however for the purposes of the statistical analysis we used one measurement per patient. We hope this guideline will facilitate the decision making on how to proceed with a child presenting with gastrointestinal symptoms in primary and secondary care.

## Discussion

We assessed the results of FCP samples from two large paediatric cohorts referred to a regional paediatric gastroenterology unit, assuming that all subsequent cases of IBD were then diagnosed in this unit. Overall, 47% of all FCP values lay > 50 μg/g and 85% lay < 250 μg/g, regardless of the underlying diagnosis. Assuming that far less than 50% of patients who undergo FCP testing will ultimately be diagnosed with a GI disease such as IBD, these data clearly highlight that the current normal reference value of 50 μg/g is not an accurate threshold in the paediatric population.

In our study, the mean FCP is significantly higher in infants (aged < 1y) when compared to older children. This finding is based only on few patients (n = 34), but still it provides further evidence that infants have higher FCP levels than children aged > 1y. Previous studies reported higher FCP levels mainly in children under the age of 6y [10–15]. In our study, the difference in the FCP level between the age groups 1-5y compared to 6-14y, was—although statistically significant—not clinically relevant (41 vs. 47 μg/g).

Previous studies have provided inconsistent recommendations for age-related reference values in children, along with a variable diagnostic accuracy for diagnosing IBD in children [1, 2, 21–26]. Additionally, there are promising results from prospective studies with newer tests like Calgranulin C for the diagnostic workup of IBD [24, 25]. Whilst our retrospective data analysis cannot provide an accurate new reference range for FCP in each age group, it provides further evidence that a general increase of the FCP cut-off to at least 250 μg/g in children aged 0-16y appears appropriate [9, 18, 26]. This is due to the fact that (i) 85% of all FCP values lay < 250 μg/g (including healthy children and children with an underlying GI condition) and (ii) only 7 children with IBD (= 6.7% of children with IBD) had an FCP of < 250 μg/g and they all showed at least one matching symptom suggestive of IBD and/or abnormal blood tests.

In the group referred to us for possible IBD solely because of an elevated FCP just above 50 μg/g, 24% had been diagnosed with IBD subsequently. The low occurrence of IBD is even more evident in the subgroup with an FCP of 50–250 μg/g who were referred solely because of their FCP level: only four patients (2.8%) had IBD. This is most obviously explained by the fact that an elevated FCP alone is a weaker indicator of IBD than appropriate symptoms accompanied by an elevated FCP [18]—especially in primary and secondary care, where the prevalence of IBD is low.

Only nine out of ninety-six patients with IBD (= 8.6% of all children with IBD) had a FCP of < 600 μg/g at diagnosis, with five patients (56%) being referred due to their symptoms and/or blood test results, and not because of their FCP level. All nine patients showed matching symptoms suggestive of IBD (red flag symptoms) or abnormal blood test results. We found that if the FCP in a child—seen at this point by a paediatric gastroenterologist—is < 600 μg/g, IBD is unlikely in the absence of matching symptoms suggestive of IBD or in the absence of abnormal blood tests. This message is supported by the ROC/AUC analysis for the IBD probability, as it revealed a corresponding FCP cut-off value of 600 μg/g. This was expected as most of our patients with IBD had FCP ≥ 600 μg/g. In view of these findings, a more useful advisory for paediatric gastroenterologists recommending a diagnostic endoscopy in children would be an FCP of > 600 μg/g in the presence of appropriate symptoms and/or abnormal blood tests.

## Conclusion

Our findings further support the recommendation that FCP levels of 250 μg/g or more are required in children with GI symptoms suggestive of IBD to refer to a paediatric gastroenterologist. Further prospective studies are needed to validate this statement. For the paediatric gastroenterologists, we advise to be aware that an FCP result below 600 μg/g makes a diagnosis of IBD less likely, even in presence of symptoms. In addition, GPs and General Paediatricians should remain alert to the frequent high levels of FCP in children aged < 6y. Therefore, a flowchart for guidance on how to proceed with a child with gastrointestinal symptoms in primary and secondary care is provided. We take the view that there is an urgent need for an ESPGHAN guideline on the use of faecal Calprotectin.

## Supporting information

S1 File Data set of all children. (CSV)

S2 File Data set of all children referred. (CSV)
